# Using Social Media to Help Understand Patient-Reported Health Outcomes of Post–COVID-19 Condition: Natural Language Processing Approach

**DOI:** 10.2196/45767

**Published:** 2023-09-19

**Authors:** Elham Dolatabadi, Diana Moyano, Michael Bales, Sofija Spasojevic, Rohan Bhambhoria, Junaid Bhatti, Shyamolima Debnath, Nicholas Hoell, Xin Li, Celine Leng, Sasha Nanda, Jad Saab, Esmat Sahak, Fanny Sie, Sara Uppal, Nirma Khatri Vadlamudi, Antoaneta Vladimirova, Artur Yakimovich, Xiaoxue Yang, Sedef Akinli Kocak, Angela M Cheung

**Affiliations:** 1 Faculty of Health, School of Health Policy and Management York University Toronto, ON Canada; 2 Vector Institute Toronto, ON Canada; 3 Department of Medicine and Joint Department of Medical Imaging, University of Toronto Toronto, ON Canada; 4 Hoffmann-La Roche Ltd Toronto, ON Canada; 5 Electrical and Computer Engineering, Queen’s University Kingston, ON Canada; 6 Manulife Toronto, ON Canada; 7 Deloitte Toronto, ON Canada; 8 TELUS Health Montreal, QC Canada; 9 Department of Pediatrics, Faculty of Medicine, University of British Columbia Vancouver, BC Canada; 10 Roche Information Solutions San Francisco, CA United States; 11 Hoffmann-La Roche Ltd Munich Germany; 12 University Health Network Toronto, ON Canada

**Keywords:** long COVID, post–COVID-19 condition, PCC, social media, natural language processing, transformer models, bidirectional encoder representations from transformers, machine learning, Twitter, Reddit, PRO, patient-reported outcome, patient-reported symptom, health outcome, symptom, entity extraction, entity normalization

## Abstract

**Background:**

While scientific knowledge of post–COVID-19 condition (PCC) is growing, there remains significant uncertainty in the definition of the disease, its expected clinical course, and its impact on daily functioning. Social media platforms can generate valuable insights into patient-reported health outcomes as the content is produced at high resolution by patients and caregivers, representing experiences that may be unavailable to most clinicians.

**Objective:**

In this study, we aimed to determine the validity and effectiveness of advanced natural language processing approaches built to derive insight into PCC-related patient-reported health outcomes from social media platforms Twitter and Reddit. We extracted PCC-related terms, including symptoms and conditions, and measured their occurrence frequency. We compared the outputs with human annotations and clinical outcomes and tracked symptom and condition term occurrences over time and locations to explore the pipeline’s potential as a surveillance tool.

**Methods:**

We used bidirectional encoder representations from transformers (BERT) models to extract and normalize PCC symptom and condition terms from English posts on Twitter and Reddit. We compared 2 named entity recognition models and implemented a 2-step normalization task to map extracted terms to unique concepts in standardized terminology. The normalization steps were done using a semantic search approach with BERT biencoders. We evaluated the effectiveness of BERT models in extracting the terms using a human-annotated corpus and a proximity-based score. We also compared the validity and reliability of the extracted and normalized terms to a web-based survey with more than 3000 participants from several countries.

**Results:**

UmlsBERT-Clinical had the highest accuracy in predicting entities closest to those extracted by human annotators. Based on our findings, the top 3 most commonly occurring groups of PCC symptom and condition terms were systemic (such as *fatigue*), neuropsychiatric (such as *anxiety* and *brain fog*), and respiratory (such as *shortness of breath*). In addition, we also found novel symptom and condition terms that had not been categorized in previous studies, such as *infection* and *pain*. Regarding the co-occurring symptoms, the pair of *fatigue* and *headaches* was among the most co-occurring term pairs across both platforms. Based on the temporal analysis, the neuropsychiatric terms were the most prevalent, followed by the systemic category, on both social media platforms. Our spatial analysis concluded that 42% (10,938/26,247) of the analyzed terms included location information, with the majority coming from the United States, United Kingdom, and Canada.

**Conclusions:**

The outcome of our social media–derived pipeline is comparable with the results of peer-reviewed articles relevant to PCC symptoms. Overall, this study provides unique insights into patient-reported health outcomes of PCC and valuable information about the patient’s journey that can help health care providers anticipate future needs.

**International Registered Report Identifier (IRRID):**

RR2-10.1101/2022.12.14.22283419

## Introduction

Postacute sequelae of SARS-CoV-2, known as post–COVID-19 condition (PCC) or colloquially, long COVID, are broadly defined as delayed recovery from infection with SARS-CoV-2. PCC can occur following severe, mild, or even asymptomatic SARS-CoV-2 infection [1]. Patients with PCC experience lingering or episodic symptoms for greater than 12 weeks or 3 months after acute infection [1,2]. Despite a growing interest in characterizing clinical manifestations of PCC, no standard framework has yet been established [3,4]. Symptoms of PCC are extremely heterogeneous and their assessment varies widely among studies.

Multiple pioneering efforts have investigated symptoms of PCC for hospitalized individuals, representing the minority of people with COVID-19 [5-7]. An extensive patient-led survey has been conducted in an outpatient setting to explore the symptoms of PCC over 7 months [3]. The survey analyzed numerous symptoms of PCC from 3762 confirmed (or suspected) patients with COVID-19 and attributed them to 10 organ systems. To our knowledge, this study is one of the most highly cited papers providing insights into PCC for researchers and clinicians. However, as the authors note, a limitation of the study is the existence of a sampling bias toward patients with PCC who opted to participate in the study. The authors recommend greater outreach with diverse groups of patients is needed to counter sample bias and better characterize the PCC phenomenon. This shortcoming motivates the proposed social medial approach to improve understanding of PCC by filling the information gaps in a more diverse patient population.

The rise of social media platforms has provided researchers and public agencies an unprecedented opportunity to gain insight into personal and population health experiences outside traditional health care settings [8-11]. Global content on social media platforms is consistently expanding with users expected to increase to 4.4 billion individuals by 2025 [12]. With an appropriate data analytics approach, these data have proven useful in generating insights into emerging health conditions, as seen with Ebola virus [13], Zika virus [14], and foodborne disease [15]. Numerous studies have used Twitter and Reddit posts as valuable resources for studying public health measures, the evolution of new medical conditions [16-18], and exploring populations’ health during and after COVID-19 [19-23].

A close examination of prior social media studies demonstrates that generating valuable insights (ie, clinical symptoms) from social media platforms requires complex and well-designed natural language processing (NLP) approaches [15,24,25]. NLP, so far, has had a significant impact on COVID-19 research and response efforts; NLP techniques have been used to predict the initial spread of COVID-19 quicker than public health [26], extract COVID-19 content from social media posts [24,27], and detect COVID-19 misinformation videos on YouTube [28]. In a recent study, NLP was implemented to characterize a broad set of COVID-19 signs and symptoms from medical records, with enhanced detail and timeliness [29]. Altogether, these findings provide clear evidence that NLP can strengthen PCC surveillance and help researchers and public health officials understand the public perceptions and attitudes toward the long-term impact of COVID-19 infection on physical and psychological health. Despite all these promising points, adopting and integrating these types of patient-generated data into broader health research and services requires a comprehensive evaluation of the data and the NLP model. This evaluation task is laborious due to the lack of a “ground truth” from social media data, requiring the pooling of resources from related literature and engaging subject matter experts [18,30,31].

**Figure 1:**
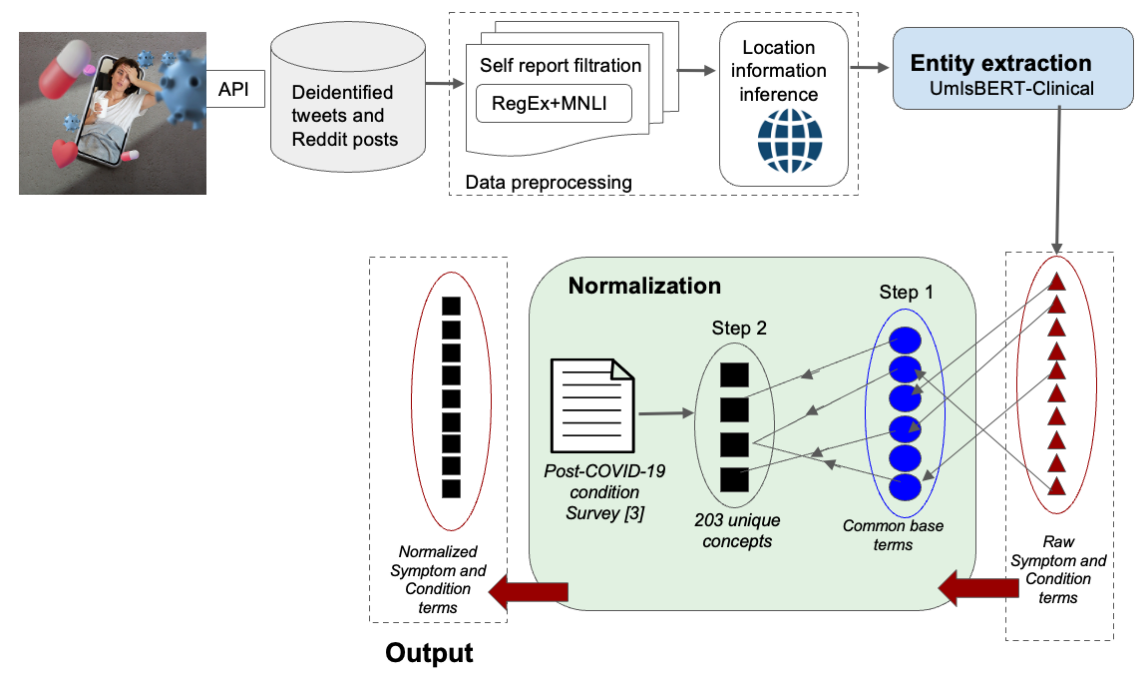
Illustration of implementation of an end-to-end natural language processing pipeline for extracting information from user-reported experiences in the social media platforms Twitter and Reddit. The data preprocessing step in the pipeline includes self-report extraction and location information inference. Next in the pipeline is the extraction and 2-step normalization of post–COVID-19 condition terms. UmlsBERT-Clinical is used for term extraction tasks. The first step of normalization involves mapping terms to their common base forms. The second step of normalization involves mapping from base forms to unique concepts derived from the post–COVID-19 condition survey. API: application programming interface; MNLI: multi-genre natural language inference; RegEx: regular expression approach.

In response to the emergence of PCC, we developed an NLP pipeline as shown in Figure 1 to facilitate extracting information from user-reported experiences in social media platforms [32]. In this study, we examined the validity and effectiveness of our NLP pipeline to provide insights into patient-reported PCC-related health outcomes across 2 popular social media platforms, Twitter and Reddit. In doing so, we extracted symptoms and conditions and estimated their occurrence frequency. We compared the outputs with human annotations and highly used clinical outcomes grounded in the medical literature. Lastly, we tracked occurrences of symptom and condition terms over time and geographies to explore the pipeline’s potential to be used as a surveillance tool reflecting users’ opinions and experiences.

## Methods

### Data Collection

We used application programming interfaces (APIs) to collect data from the 2 social media platforms for the period from 2019 until 2021. We hashed all usernames and removed URLs through the deidentification process. To further pseudonymize the data, we transformed special characters in the tweets or Reddit posts to lowercase and extracted contractions. Multimedia Appendix 1 and our previous work describe more details about the data collection and preprocessing steps [32].

For Twitter searches, we chose relevant hashtags (eg, #longcovid, #covidlong, or #longhauler) and words included in tweets (eg, *long-hauler*, *chronic symptoms*, and *long-term effects*). We excluded retweets, replies, quotes, and nullcast from the data set, as well as any tweets that were not in English. The list of hashtags is shown in Table S1 in Multimedia Appendix 1. Our Twitter data set’s average character length and word count were 133 and 23, respectively. For the Reddit data, we targeted specific subreddits such as r/covidlonghaulers that were appropriate to the PCC topic. In contrast to the short length of tweets (up to 280 characters), Reddit posts can have up to 40,000 characters, and the longest Reddit post in our data set had 17,060 characters.

### Data Preprocessing

#### Self-Report Extraction

For this study we were primarily interested in posts containing self-reported medical symptoms. We therefore excluded individual posts in our corpus with other purposes at the time of posting, such as disseminating news and sharing ideas and opinions. We used a combination of the regular expression approach (RegEx) and transformer-based bidirectional encoder representations from transformers (BERT) classifier to distinguish between posts of self-reports (explaining personal health status) and other posts, including opinions or news reports. The RegEx approach relies on personal pronouns, as shown in Table 1. The classifier, which we call MNLI classifier, is a natural language inference model—the COVID-Twitter BERT (version 2), fine-tuned on the MultiNLI data set [24]. The classifier annotates posts as either self-report (annotated as “1”) or not (annotated as “0”) and poses the candidate labels (eg, *my experience*) as either “premise” or “hypothesis.” We ran the RegEx model and the combination of RegEx and MNLI classifier (RegEx+MNLI classifier) on the entire Twitter data set. We saved the scores, with the averaged performances shown in Table 2. The standalone RegEx filter outperformed the RegEx+MNLI classifier without substantial loss in precision, as reflected by the *F*_1_-score (Table 2). Although the proportion of self-reports is much higher on Reddit, the same self-filtration approach was applied to Reddit posts for consistency.

**Table 1:** Self-report extraction. Pronouns and respective regular expression (RegEx) were used for extraction of self-reports from social media posts.

Pronoun captured	Regular expression code
I	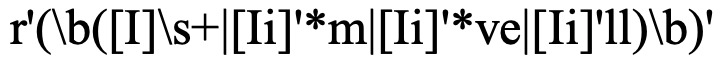
Me	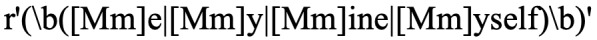

**Table 2:** Self-report extraction. The performance results of the self-report filters on the Twitter data set—RegEx+MNLI classifier is the combination of RegExa and COVID-Twitter BERT (version 2) fine-tuned on the MultiNLI data set.

Approach	Accuracy	Precision	Recall	*F*_1_
RegEx^a^+MNLI^b^ classifier	0.83^c^	0.79^c^	0.71	0.74
RegEx	0.78	0.75	0.83^c^	0.76^c^

^a^RegEx: regular expression.

^b^MNLI: natural language inference model.

^c^The highest value in each column.

#### Location Information Inference for Twitter Data

For the Twitter data set, we used the open source Nominatim API [33] to infer detailed location information such as city, region, and country from user-defined location information attached to tweets. The API uses OpenStreetMap (OpenStreetMap Foundation) data to make predictions. In cases where user-defined location information could allude to multiple locations (eg, London is a city in both Canada and England), Nominatim API returns the most likely location based on its criteria. For the Reddit data set, location information was not available.

### Extraction and Normalization of PCC Symptoms and Conditions

Transformer-based BERT models were mainly used to extract and normalize PCC symptoms and conditions. Specifically, we used and compared 2 named entity recognition (NER) models, including UmlsBERT-Clinical [32] and the Stanza clinical NER (Stanza-Clinical). UmlsBERT-Clinical model is an UmlsBERT NER fine-tuned on the n2c2 (2010) data set. Stanza-Clinical is a publicly available NER from the Stanford NLP group. Given the BERT models’ input length limit, we used rolling windows to segment Reddit posts into smaller chunks with less than 1024 characters.

Following the symptom and condition term extraction task, we implemented a 2-step normalization task, as shown in Figure 1, to analyze our findings, reduce inflectional forms, and compare them with existing works. The 2-step normalization task maps extracted terms to unique concepts in a standardized terminology. In the first step of normalization, we mapped each extracted raw term into its common base form. For instance, *my tiredness*, *real tiredness*, *very tired,* and *chronic tiredness* were normalized to *tired.* For this task, all extracted terms (eg, *my* *tiredness*) were tokenized, tagged, and clustered into nouns (eg, *tired*), pronouns (eg, *my*), or suffixes (eg, *ness*). Then, common base forms for terms were built through a manual review of all the nouns. In the second step of normalization, we transformed the terms in their common base form into their corresponding unique concept in a standardized terminology, for example, *tired* is mapped to *fatigue*. The standard terminologies were derived from a highly cited and used PCC research paper which gathered 203 symptoms from 3762 patients with PCC through a web-based survey [3]. We, therefore, used the 203 symptoms as the standardized unique concept for the second step in the normalization procedure.

The conversion between extracted raw terms from social media and either the common base form or the 203 unique concepts was done using a semantic search approach with BERT biencoders [34]. Using this approach, we first created embeddings for all the extracted raw symptom and condition terms. Then, we retrieved the top common bases or unique concepts with high semantic overlap with the raw terms at the search time. Following a manual review of the retrieved pair, we set a cutoff threshold where pairs with similarity scores greater than the threshold were stored as the match and included in our analysis. For the rest of this paper, *mapped* terms refer to the raw symptom and condition terms mapped to their common base in the first step of the normalization process. In addition, *normalized* terms refers to normalized symptom and condition terms further transformed to the 203 standardized unique concepts derived from 3762 patients with PCC.

### Evaluation

To determine how good the BERT models are at extracting the symptom and condition terms, we created a human-annotated corpus from Twitter. We established a proximity-based score to measure potential overlap. Our in-house human-annotated Twitter corpus includes 200 randomly sampled tweets annotated by 4 trained annotators. The proximity-based score was calculated by dividing the intersection of extracted entities by the union of extracted terms, with duplicated extracted entities removed for annotators and the model. The closer the proximity metric to 0, the closer the model’s predictions to the human-annotated benchmark. Validity and reliability of the extracted and normalized symptoms, a subset of symptom and condition terms, were evaluated compared with a web-based survey including 3762 participants from 56 countries [3,35,36].

### Ethical Considerations

Several ethical considerations were examined during this study. The initial consideration pertained to whether the ethical standards were met when using social media data [37]. It is important to note that Ethics approval was not pursued for this study. In accordance with our institution's ethical perspective, the analysis of deidentified public data for trend and insight generation is deemed acceptable. This is on the condition that no individual-level data is disclosed and the risk of reidentification remains minimal. Deidentification measures were implemented at the beginning of the data set development process in accordance with our institution's viewpoint, and the updating of the data set made available for analysis has been restricted. As a measure to protect privacy, hashing was applied to all usernames and mentions during the deidentification process. Moreover, our research does not involve any direct interaction with social media users. Additionally, all the project participants have signed a data user agreement that restricts access and usage of the data for the sole purpose of use in this scientific research and not for any other purpose.

## Results

### Data Overview

Table 3 lists the statistics of the English-language posts collected from Twitter and Reddit. In addition to posts, we gathered the timestamp, geographical coordinates (if available), user’s location (user-defined), and user’s profile description for each tweet. A total of 107 countries were represented in our Twitter sample; most respondents tweeted from the United States (4850/10,938, 44.3%), followed by the United Kingdom (4316/10,938, 39.4%) and Canada (631/10,938, 5.8%).

**Table 3:** Statistics of Twitter and Reddit data.

Platform	Period	Posts, n			Raw terms^a^, n				Total^b^ mapped terms^a^, n		Total^b^ normalized terms^a^, n
		Before filter^c^	After filter^c^	Posts^d^				Unique^e^		Total^c^	
Twitter	August 2019 to June 2021	466,651	84,621	28,202		22,451	52,806	33,175		26,247	
Reddit	July 2020 to September 2021	191,526	129,917	128,820		92,816	357,887	243,342		209,193	

^a^Extracted symptom and condition terms using UmlsBERT

^b^Total denotes the total occurrence counts of extracted symptom and condition terms.

^c^The filter refers to the self-report filter.

^d^Posts denotes the count of posts with at least 1 extracted symptom and condition term.

^e^Unique denotes the counts of unique extracted symptom and condition terms.

### Evaluation of Extraction of Symptom and Condition Terms

Table 4 compares the performance of UmlsBERT-Clinical and Stanza-Clinical for the entity extraction task from Twitter data. Both models’ performances are compared against human annotators regarding the proximity score. As shown in the table, we also included entity extraction results by the data augmentation approach UMLS MetaMap (+American Medical Informatics Association; AMIA) introduced in our earlier work [32]. UMLS MetaMap (+AMIA) uses the MetaMapLite tool to extract entities associating with UMLS’ concept unique identifiers and augments the results with a manually annotated data set consisting of clinical concepts and colloquial expressions (eg, *brain fog*) from tweets. Based on the results, UMLS MetaMap (+AMIA) tends to capture more entities than human annotators; however, some may not be as relevant or provide sufficient insight to experts. Stanza tends to capture fewer entities than human annotators. Consequently, there is the risk of missing information that subject matter experts may consider relevant. UmlsBERT-Clinical has the lowest sum of absolute values for the proximity-based evaluation metric (0.28), indicating predictions closest to those extracted by human annotators from the sample. Hereafter, the rest of the analysis is based on using UmlsBERT-Clinical for the extraction task due to its better overall performance than Stanza.

**Table 4:** Proximity score. The comparison results of the proximity score of human evaluation on 200 tweets were identified by trained annotators with outputs from UmlsBERT-Clinical, Stanza-Clinical, and MetaMap +AMIA.^a^ Annotators 3 and 4 have medical backgrounds. The comparison values are based on the proximity-based evaluation metric, defined as the difference between the annotator’s and model’s match counts. The closer the proximity metric to 0, the closer the model’s predictions to the ground truth (human annotator).

	UmlsBERT-Clinical [32]	MetaMap+AMIA [32]	Stanza-Clinical
Annotator 1	−0.13	−0.46	0.33
Annotator 2	−0.02	−0.29	0.33
Annotator 3	0	−0.31	0.51
Annotator 4	0.13	−0.17	0.48
Sum of the absolute values	0.28	1.23	1.65

^a^AMIA: American Medical Informatics Association

### Occurrence Frequency Estimation at Any Point in Time

#### Overview

**Figure 2:**
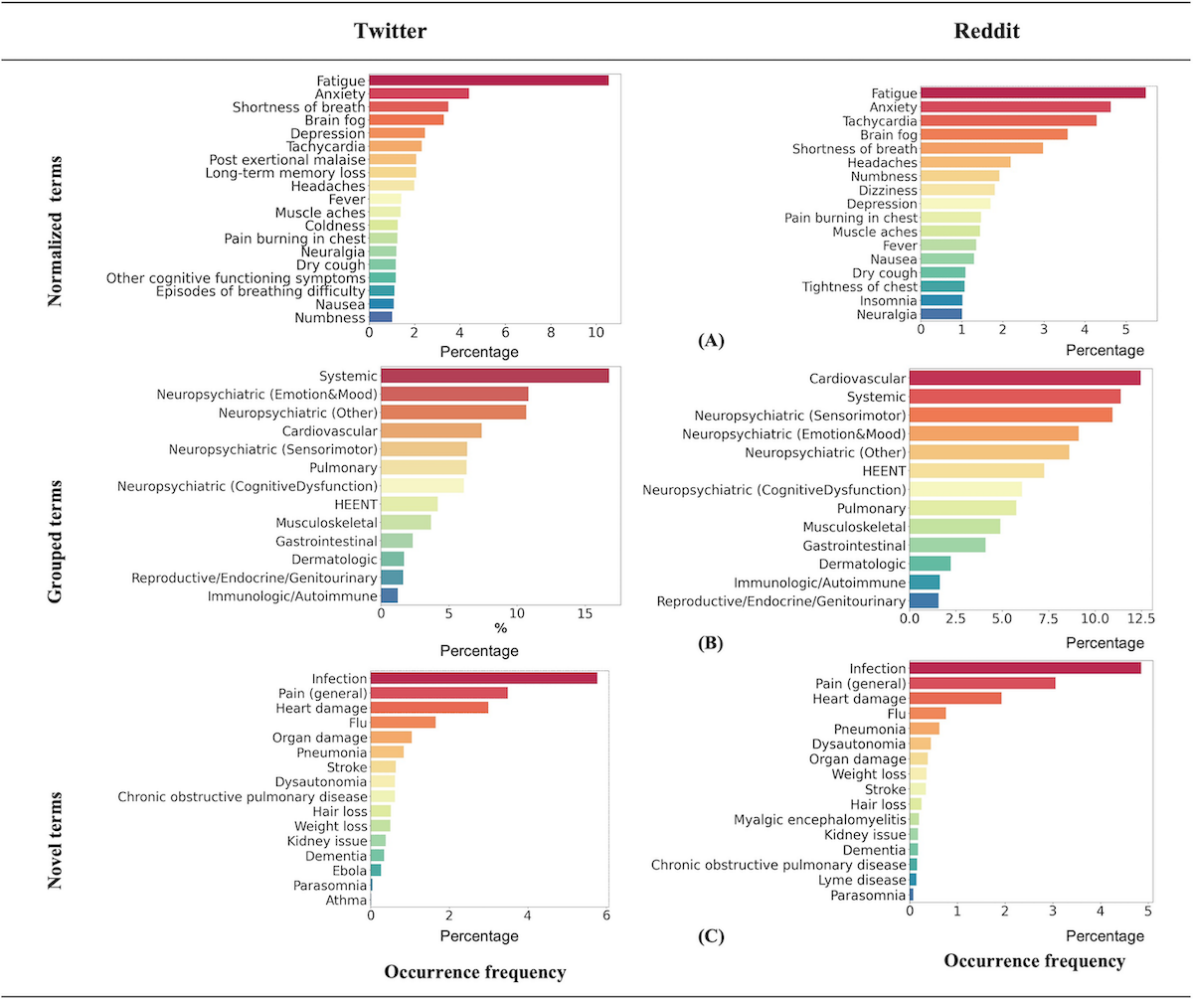
The occurrence frequency of the most prevailing extracted symptom and condition terms in Twitter and Reddit data with occurrence frequency greater than 1% (n>350 for Twitter, and n>4000 for Reddit). Normalized terms are the raw terms that were normalized (after a 2-step normalization process, as shown in Figure 1) to the 203 standardized unique concepts derived from a web-based survey of 3762 patients with post–COVID-19 condition [3]. For instance, “my tiredness” is normalized into “fatigue.” Grouped terms are the normalized terms that were further categorized based on the affected organ system established by Davis et al [3]. Novel terms are the mapped terms that we had not normalized to the 203 standardized unique concepts because they were neither reported nor categorized in the survey study [3]. HEENT: head, eyes, ears, nose, and throat.

The occurrence frequency of normalized symptom and condition terms at any point in time is shown in Figure 2. Figure 2A and Figure 2B depict the occurrence frequency of the normalized terms, and Figure 2C illustrates the occurrence frequency of mapped terms. The normalized terms were further categorized by the affected organ systems, similar to the survey study [3], and the aggregated occurrence frequency per each category is shown in Figure 2B.

#### Comparison of Social Media to the Survey Study

Systemic and neuropsychiatric symptoms were evidenced as the top-occurring symptoms in our study and the survey study. Fatigue (Twitter: 3434/33,175; Reddit: 3486/243,342; so n~3400, 10.3%) appeared as the most frequent symptom on both Twitter and Reddit. On Twitter (n=33,175), the top 5 most occurring symptoms also included anxiety (n=1433, 4.8%), shortness of breath (n=1136, 3.5%), brain fog (n=1072, 3.4%), and depression (n=803, 2.7%), whereas for Reddit (n=243,342), they also included anxiety (n=3984, 4.6%), tachycardia (n=3016, 4.3%), brain fog (n=3521, 3.6%), and shortness of breath (n=1889, 2.7%). This observation is in line with the survey study [3], where fatigue, breathing issues, and cognitive dysfunction (eg, depression and anxiety) were reported by patients as the top 3 most debilitating symptoms. Other terms, including *headaches*, *dizziness*, *pain*, *burning in the chest*, *fever*, *nausea*, *dry cough*, and *neuralgia* appeared in the top 20 occurring terms in both Twitter and Reddit data albeit with slightly different prevalence (Figure 1A). *Immunologic/autoimmune*, *dermatology*, and *reproductive/genitourinary/endocrine* were the lowest 3 categories of terms across all 3 sources—Twitter, Reddit, and the survey study.

#### Discovery of Novel Conditions

Novel symptom and condition terms shown in Figure 2C are neither reported nor categorized in the survey study [3]. Among novel terms, *infection* and *pain* are the top 2 reported conditions. Other terms, including *flu*, *organ damage*, *hair loss*, *weight loss*, *dementia*, *parasomnia*, *pneumonia*, *dysautonomia*, *kidney issues*, and *chronic obstructive pulmonary disease* were among the top 1% (Twitter: n=500; Reddit: n=700) of reported terms.

### Co-Occurrence Frequency Estimation at Any Point

**Figure 3:**
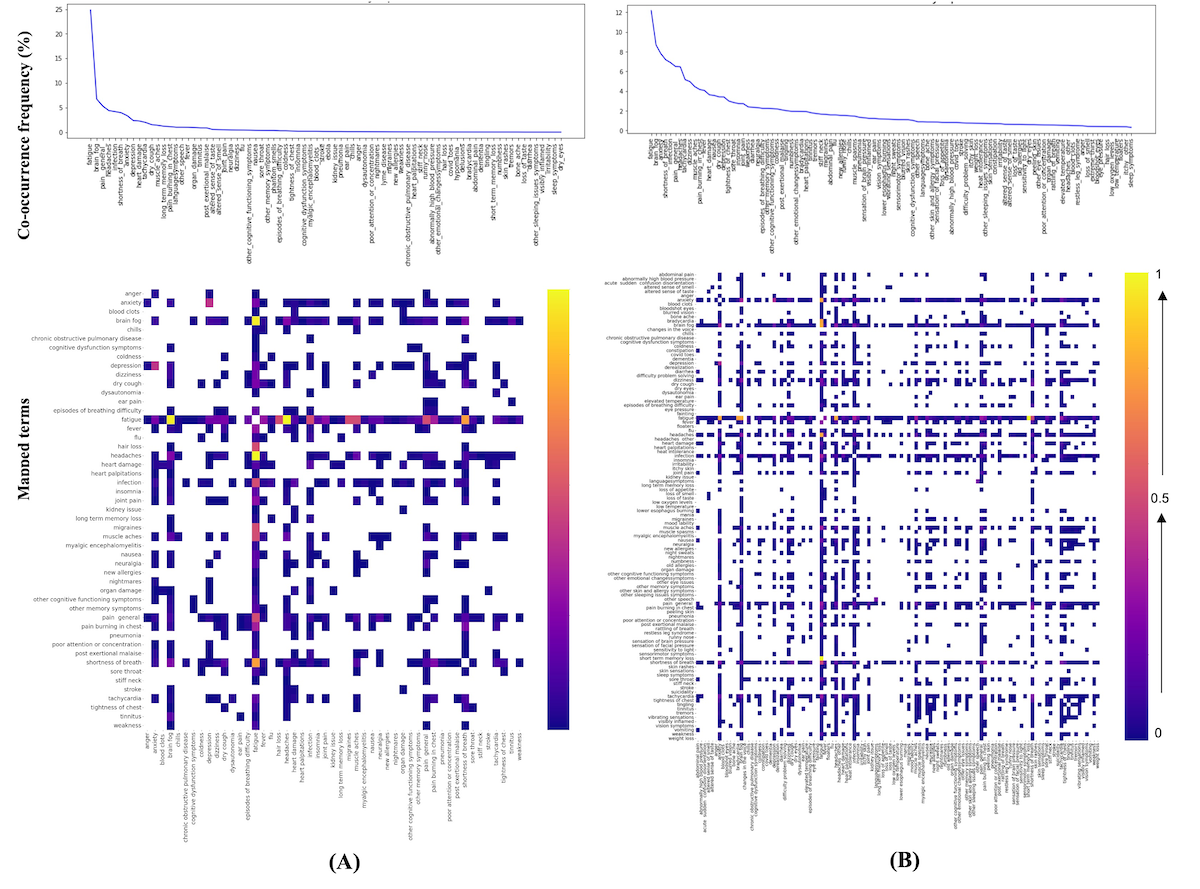
Co-occurrence frequency of normalized post–COVID-19 condition terms in Twitter (A) which is higher than 50% and Reddit (B) which is higher than 10% data. Higher values are shown by the intensity of pink and blue shading. Normalized terms are the raw terms that were normalized (after a 2-step normalization process, as shown in Figure 1) to the 203 standardized unique concepts derived from a web-based survey of 3762 patients with post–COVID-19 condition [3]. For instance, “my tiredness” is normalized into “fatigue”. Please see Multimedia Appendix 2 for a larger version.

Figure 3 shows how often pairs of PCC-normalized symptom and condition terms co-occur in both Twitter and Reddit. As expected, the co-occurrence map is more “dense” for the Reddit data (Figure 3A) than the Twitter data (Figure 3B). Since Reddit posts are significantly longer than tweets, they contain more contextual information and repeated symptom and condition terms. Based on the results, the pair of *fatigue* and *headaches* was among the most co-occurring terms across both platforms. In addition, for Twitter data, the pairs of *fatigue* and *shortness of breath*, *fatigue* and *migraines*, *fatigue* and *general pain*, *fatigue* *and hair loss*, *fatigue* and *infection*, *brain fog* and *fatigue*, and *depression* and *anxiety* co-occur more commonly than other terms; for Reddit, common symptom and condition pairs include *fatigue* and *bradycardia*, *fatigue* and *anxiety*, and *fatigue* and *short term memory loss*.

### Spatio-Temporal Frequency Estimation

**Figure 4:**
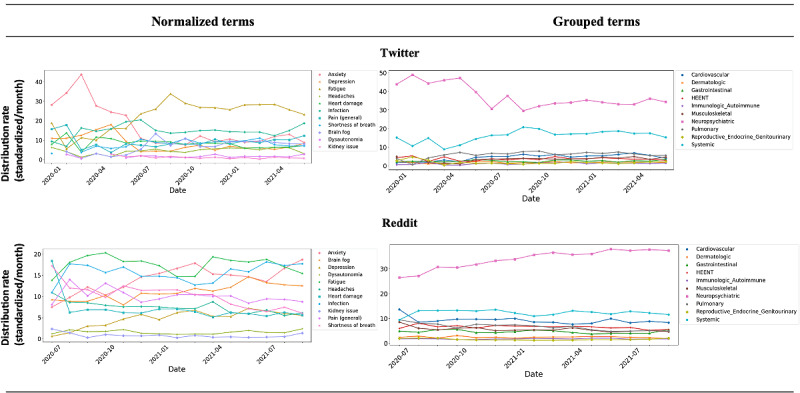
The distribution rate of normalized and grouped post–COVID-19 condition terms over time; the rates are standardized per month.

The distribution of normalized symptom and condition terms (standardized per month) over time is shown in Figure 4 for Twitter and Reddit data. The incidence of the neuropsychiatric symptom and condition terms is dominant, followed by the systemic category, across both social media platforms. On a more granular level, *fatigue*, *anxiety*, and *infections* were the most prevalent terms reported. Our findings indicate that the predominance of terms varied over time, where for Twitter, *anxiety* was dominant through June 2020, and afterward, *fatigue* was the most commonly reported symptom. *Infection* has been reported by users as a persistent condition for the entire period. On Reddit, for most periods, *fatigue* and *infection* were more dominant than *anxiety*.

**Figure 5:**
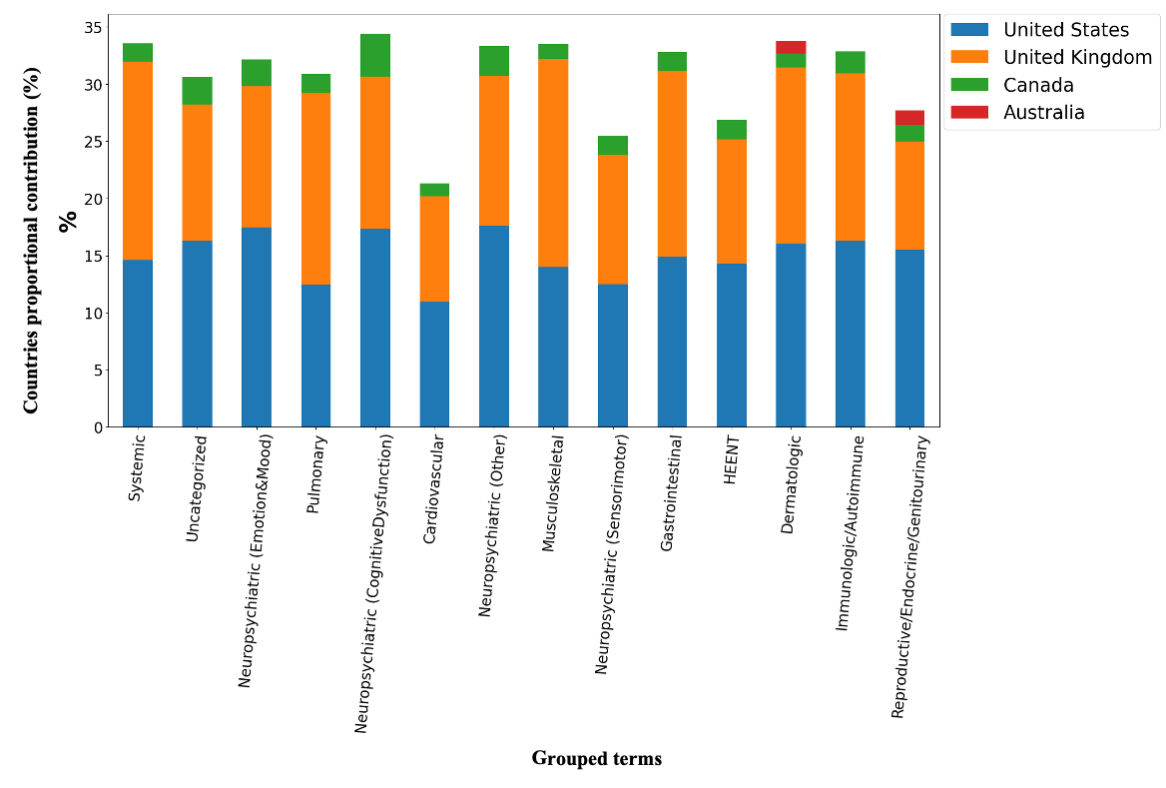
The proportional contribution (n=10,878, 41%) of the top 4 countries (the United States, United Kingdom, Canada, and Australia) to each group's occurrence frequency of symptom and condition terms. The proportions are measured as a percentage of frequency group-related terms per each country group divided by the total count of terms in that group. HEENT: head, eyes, ears, nose, and throat.

Based on our spatial analysis performed on Twitter data, among all the normalized symptom and condition terms (26,247 terms aggregated across all tweets, as shown in Table 3), 41% (n=10,878) included location information. The 41% (n=10,878) were spread across 62 countries, whereby the United States (n=4850, 15%), United Kingdom (n=4316, 13%), and Canada (n=631, 2%) were the top 3 for self-reporting of symptoms related to PCC (full details are listed in Multimedia Appendix 1). The other 59 countries reported fewer than 1% (n<251) of the symptom and condition terms and were excluded from our analysis. Figure 5 indicates the proportional contribution of the top 4 reporting countries to the total occurrence frequency of symptom and condition terms normalized to unique concepts in KB and grouped by the affected organ system.

## Discussion

### Overview

The overarching goal of this study was to highlight the possibility of gaining insight into the patient’s experience of PCC using social media and NLP approaches. User-generated social media data provide a rich yet challenging source of information about the patient's journey outside the health care setting. Significant limitations remain concerning recognizing a patient’s lived experience instead of opinion and aligning common vernacular to recognized medical terminology. However, our study has made progress toward narrowing the data quality gap and evaluating the validity and reliability of social media-driven outcomes regarding PCC using state-of-the-art NLP approaches. Our results suggest that there is value in the learnings and methodologies outlined in this study to gather insights from patient-reported outcomes on social media platforms.

### Principal Findings

Our transformer-based entity extraction tool, clinical UmlsBERT, outperformed Stanza and UMLS MetaMap (+AMIA) to extract symptom and condition terms from both Twitter and Reddit. Our results confirm the previous view that augmenting contextual embeddings with expert domains from a knowledge base outperforms domain-specific models on common named-entity recognition inside and outside health care settings [25,38]. The outcome of our NLP pipeline, mapped and normalized symptom and condition terms, was comparable with the outcomes of peer-reviewed papers relevant to PCC symptoms [3,35,36,39]. Our analysis confirms prior findings that PCC is a multisystemic condition affecting multiple organ systems. Our study showed that *fatigue*, *brain fog*, *anxiety*, and *shortness of breath* are the most commonly occurring groups of terms for PCC symptoms on Twitter and Reddit. This aligns with the primary discoveries of recent studies [3,35,36,39], where the top 3 most debilitating symptoms listed by patients were fatigue, breathing issues, and cognitive impairment.

Our findings support the increasing interest in and confirm the need for supplementing clinical observation with user-generated data [40]. In this study, we reported the frequency of co-occurring symptom and condition terms. Based on our results, the pair of *fatigue* and *headaches* was among the most co-occurring terms on both social media platforms; to the best of our knowledge, expressing a combination of symptoms related to PCC has been rarely reported in the literature, certainly not from social media based studies. One instance of clinically derived analysis of symptom co-occurrence comes from a US-based retrospective cohort study [36] evaluating long-term symptoms in COVID-19 survivors, where they similarly observe that fatigue tends to co-occur frequently with abnormal breathing or shortness of breath in patients with PCC. The potential value of this analysis is in connecting symptom and condition terms early in illness onset with prognostic factors, such as the likelihood of developing PCC, and the severity or duration of the PCC condition.

Our study and the survey study by Davis et al [3] share another point of agreement: uncovering symptoms that are not commonly mentioned in public discussion of PCC, which we call novel symptoms and conditions. Our study revealed that users experienced unique symptoms like infection, hair loss, and weight loss, as well as reported conditions that resembled those of other illnesses such as flu, cancer, or Lyme disease.

One strength of the NLP pipeline developed in this study is its scalability, leading to high-level adaptive capacity for other social media platforms or different medical conditions. Our NLP pipeline is well-poised to scale the information extraction process from user-generated data and connect vernacular to recognized medical ontology. In addition, it enables exploring the longitudinal evolution of symptoms, which may be correlated with prevalent SARS-CoV-2 variants at the time of onset to guide insights into variation in disease course associated with different source viral strains.

### Limitations

A common issue with automating entity extraction and normalization is losing the context behind the extracted terms (in the form of independent tokens or words). Without context, it may be difficult to interpret the meaning behind these tokens. For example, the token *death* could be extracted from a personal opinion, an expression of a feeling, or a factual statement; an example of a personal statement is “I think COVID-19 increases the likelihood of death,” whereas an example of a factual statement is “My cousin died of COVID-19.” In addition, an example of symptom expression tied to the condition is “This long COVID feels like death.” Furthermore, clustering semantically similar extracted symptoms and bringing them closer to predefined standards and common medical terms increases the risk of losing context.

Another limitation of this study relates to our self-report filter. While RegExs are effective in finding posts containing first-person self-reports, they are also prone to false positives—for example, mistakenly keeping a post that voices an opinion or reports symptoms on behalf of someone else—and false negatives—for example, mistakenly discarding a tweet that excludes first-person pronouns but would still qualify as a self-report. We, therefore, considered other context-based approaches, including a fine-tuned BERT classifier. These approaches may reduce false positive and false negative rates; however, further work is needed to annotate sufficient data sets for manually fine-tuning, sweeping, and optimizing hyperparameters.

The nature of social media data and our NLP pipeline introduces bias to our findings, which could impact the reliability of our outcomes. Social media content is unlikely to represent the broader population due to demographic biases in technology uptake, barriers to access, and regional social media platform preferences. Furthermore, only a subset of users are patients who publicly share their experiences with PCC. Users’ self-reports may also be influenced by prominent opinions reinforced on the internet through news media or other social media “influencers,” which adds to sample bias. In addition to inherent bias, our analysis is further biased by including only English-language posts.

In social media, data quality is highly variable due to the use of colloquial language (eg, “I am dying”), brevity or shorthand, and grammatical and spelling errors. Tweets were first collected based on the presence of relevant hashtags and keywords (eg, *long COVID*). While this approach successfully surfaced many relevant tweets, both false positives (eg, mistakenly keeping a tweet that refers to *long haul* in the context of transportation) and false negatives (eg, mistakenly discarding a relevant tweet because it lacks or has a typo in a target hashtag or keyword) can occur. False positives could be reduced by checking for the context surrounding a match, for example, excluding tweets that refer to long-haul flights. False negatives could be reduced using advanced semantic language models beyond keyword matching, for example, classifying clinical tweets versus those that are not.

Additionally, concerning the data quality, this study lacks the ability to verify the genuineness and authenticity of users’ posts on social media. Ensuring the authenticity of users is a crucial aspect of using social media to provide insights into public health. Some potential strategies to improve the quality of social media content and exclude fake accounts and bots include implementing account verification processes, monitoring user behavior for suspicious activity, and using available ML models built to identify fraudulent activities.

### Best Practices and Future Directions

#### Overview

In this section, we would like to explain the best practices for researchers and developers interested in applying NLP to social media to facilitate information extraction tasks.

#### Generalizable Models

UmlsBERT is a transformer-based contextual model with better generalizability and reliability than traditional entity extraction models. However, in our experiments we found that these models may overfit to occurrences of specific tokens such as *vid*. As a result, at inference time, incorrect tokens may be captured. A simple solution to extend the capabilities of these models is by looking at the frequencies of captured tokens and devising simple rules to correct the errors. A simple rule-based strategy should also remove variations of the same frequently occurring tokens. Another potential solution for this task would be using symptom ontology and standard normalization procedures to facilitate comparisons between variations of the same token. Literature references and ground truths can also be referred to for this procedure, especially in the case of new illnesses wherein ontologies may not fully capture the experiences that patients are trying to express—for example, brain fog.

#### Longitudinal Analysis

Providing a longitudinal analysis of posts from the same user will enable better characterization of the evolution of symptoms over time. However, it is essential to note that this might pose challenges for ensuring privacy as, for example, the combination of posts may increase the possibility of reidentifying a user. In addition, aggregating their posts may infer illness in patients who have not consented to such an assessment.

### Conclusions

In this study, we successfully used transformer-based BERT models to extract and normalize PCC symptom and condition terms from social media platforms. We evaluated the effectiveness, validity, and reliability of NLP models through comparison with a human-annotated corpus and a web-based survey with more than 3000 participants from several countries. In summary, the outcome of the NLP models aligned and complemented the previous research regarding the occurrence and co-occurrence frequency of PCC-related symptom and condition terms. In conclusion, our findings support that social media can augment health care research by providing insights into diseases that are captured outside usual episodes of clinical care. Moreover, it promotes pandemic advance monitoring and response by enhancing the scope of information-feeding risk models. Significant challenges remain for improving the accuracy and context with which symptoms are recognized (from vernacular) and interpreted (to medical ontology), which, if resolved, would add to the overall use of the process.

## Appendix

Multimedia Appendix 1 Table S1: Twitter hashtags list and Table S2: Number of tweets per country list.

Multimedia Appendix 2 Co-occurrence frequency of normalized post–COVID-19 condition terms in Twitter (A) which is higher than 50% and Reddit (B) which is higher than 10% data. Higher values are shown by the intensity of pink and blue shading. Normalized terms are the raw terms that were normalized (after a 2-step normalization process, as shown in Figure 1) to the 203 standardized unique concepts derived from a web-based survey of 3762 patients with post–COVID-19 condition [3]. For instance, “my tiredness” is normalized into “fatigue”.

## Abbreviations

AMIA: American Medical Informatics Association
API: application programming interface
BERT: bidirectional encoder representations from transformers
NER: named entity recognition
NLP: natural language processing
RegEx: regular expression

## Data Availability

The data sets generated and analyzed during this study are available in our Github repository [41].
