# Figure Correction: Using Social Media to Help Understand Patient-Reported Health Outcomes of Post–COVID-19 Condition: Natural Language Processing Approach

**DOI:** 10.2196/55010

**Published:** 2023-12-08

**Authors:** Elham Dolatabadi, Diana Moyano, Michael Bales, Sofija Spasojevic, Rohan Bhambhoria, Junaid Bhatti, Shyamolima Debnath, Nicholas Hoell, Xin Li, Celine Leng, Sasha Nanda, Jad Saab, Esmat Sahak, Fanny Sie, Sara Uppal, Nirma Khatri Vadlamudi, Antoaneta Vladimirova, Artur Yakimovich, Xiaoxue Yang, Sedef Akinli Kocak, Angela M Cheung

**Affiliations:** 1 Faculty of Health, School of Health Policy and Management York University Toronto, ON Canada; 2 Vector Institute Toronto, ON Canada; 3 Department of Medicine and Joint Department of Medical Imaging, University of Toronto Toronto, ON Canada; 4 Hoffmann-La Roche Ltd Toronto, ON Canada; 5 Electrical and Computer Engineering, Queen’s University Kingston, ON Canada; 6 Manulife Toronto, ON Canada; 7 Deloitte Toronto, ON Canada; 8 TELUS Health Montreal, QC Canada; 9 Department of Pediatrics, Faculty of Medicine, University of British Columbia Vancouver, BC Canada; 10 Roche Information Solutions San Francisco, CA United States; 11 Hoffmann-La Roche Ltd Munich Germany; 12 University Health Network Toronto, ON Canada

In “Using Social Media to Help Understand Patient-Reported Health Outcomes of Post–COVID-19 Condition: Natural Language Processing Approach” (J Med Internet Res 2023;25:e45767) the authors noted two errors in Figure presentations of Twitter data.

In the originally published article, in Figure 2B the presentation of the occurrence frequency of Grouped terms on Twitter (the left subplot) appeared incorrectly. This subplot has been corrected and the accurate presentation of the occurrence frequency of Grouped terms on Twitter has been shown.

In the originally published article, in Figure 3A the presentation of the occurrence frequency of Mapped terms on Twitter (the lower left heatmap plot) appeared incorrectly. This heatmap plot has been corrected and the accurate presentation of the co-occurrence frequency of Mapped terms on Twitter has been shown.

The originally published versions of Figures 2 and 3 are included in Multimedia Appendix 1.

**Figure 2:**
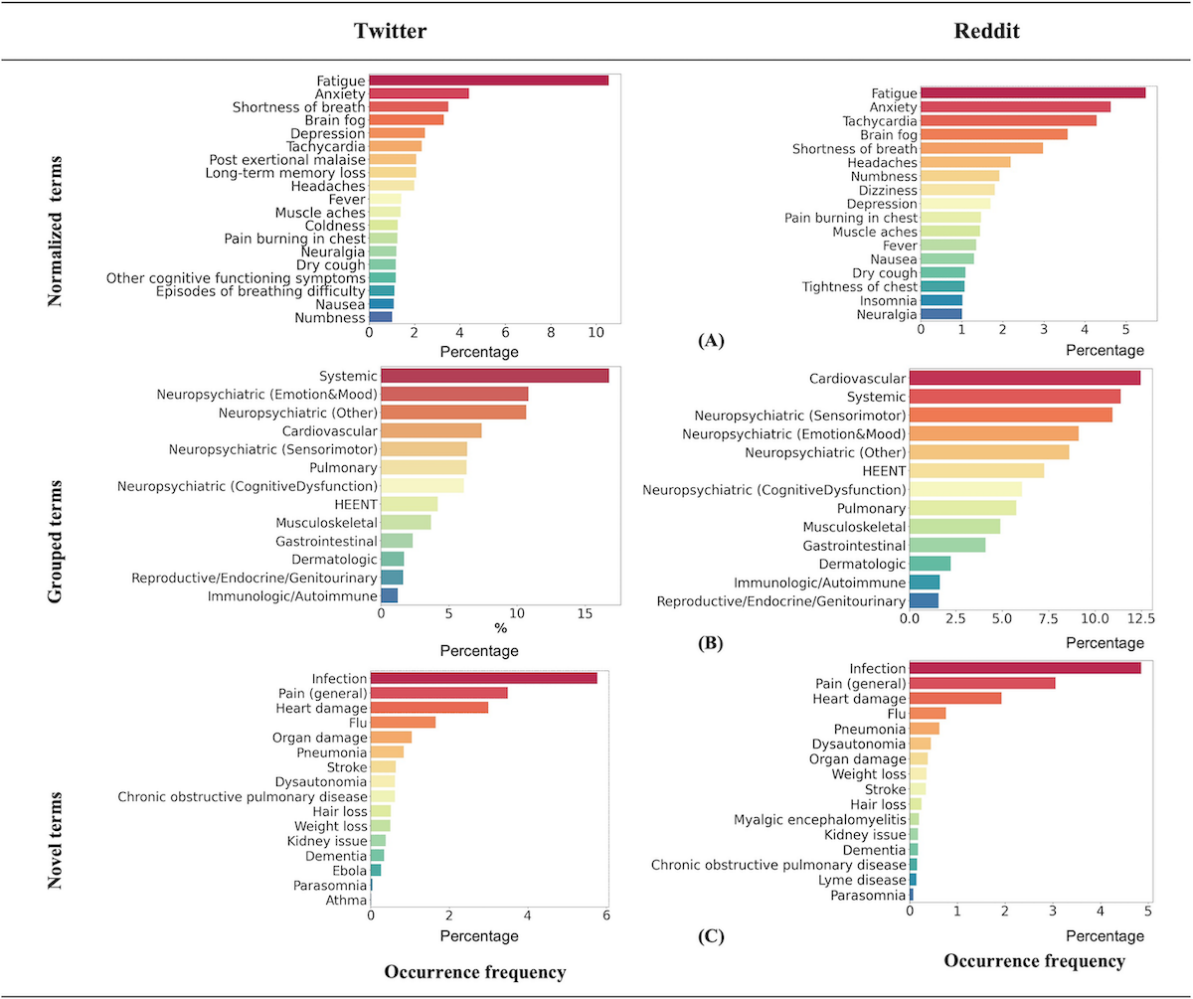
The occurrence frequency of the most prevailing extracted symptom and condition terms in Twitter and Reddit data with occurrence frequency greater than 1% (n>350 for Twitter, and n>4000 for Reddit). Normalized terms are the raw terms that were normalized (after a 2-step normalization process, as shown in Figure 1) to the 203 standardized unique concepts derived from a web-based survey of 3762 patients with post–COVID-19 condition [3]. For instance, “my tiredness” is normalized into “fatigue.” Grouped terms are the normalized terms that were further categorized based on the affected organ system established by Davis et al [3]. Novel terms are the mapped terms that we had not normalized to the 203 standardized unique concepts because they were neither reported nor categorized in the survey study [3]. HEENT: head, eyes, ears, nose, and throat.

**Figure 3:**
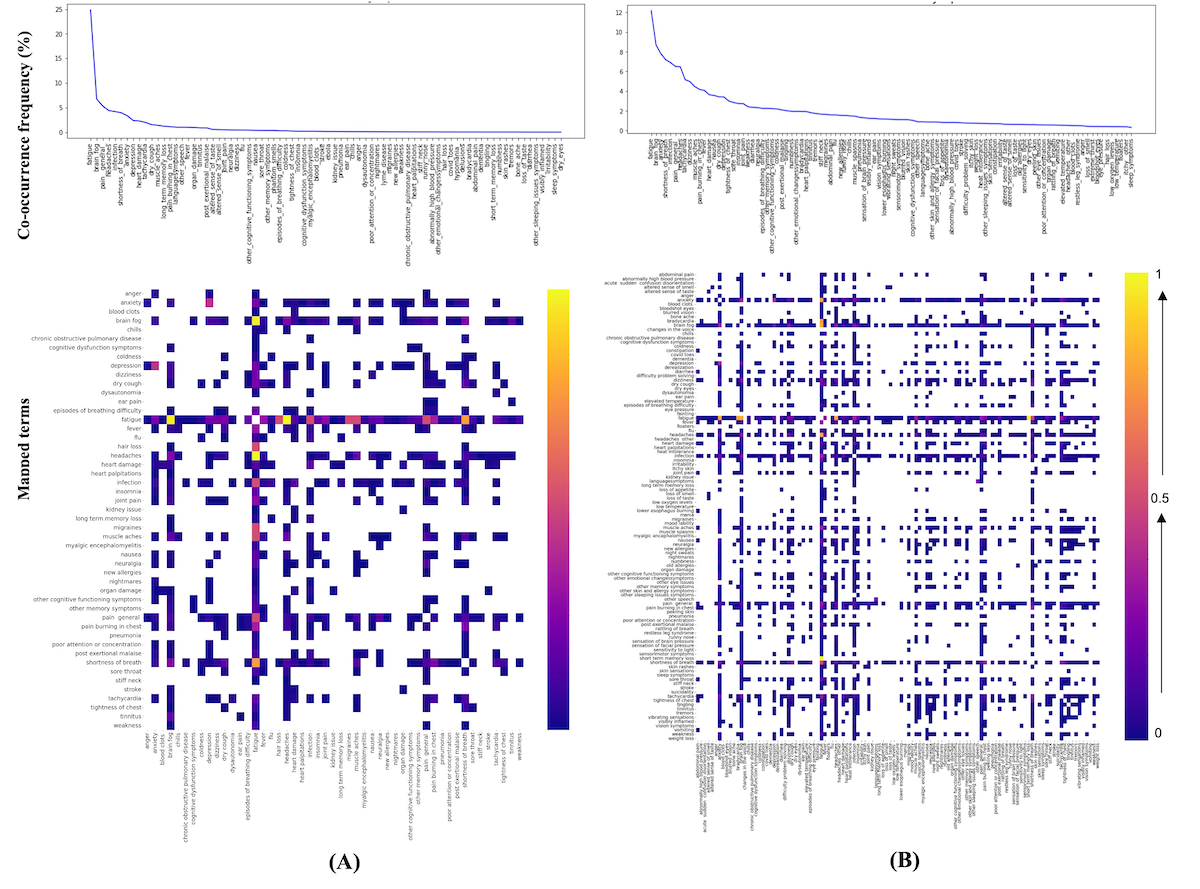
Co-occurrence frequency of normalized post–COVID-19 condition terms in Twitter (A) which is higher than 50% and Reddit (B) which is higher than 10% data. Higher values are shown by the intensity of pink and blue shading. Normalized terms are the raw terms that were normalized (after a 2-step normalization process, as shown in Figure 1) to the 203 standardized unique concepts derived from a web-based survey of 3762 patients with post–COVID-19 condition [3]. For instance, “my tiredness” is normalized into “fatigue”. Please see Multimedia Appendix 2 for a larger version.

The correction will appear in the online version of the paper on the JMIR Publications website on December 8, 2023 together with the publication of this correction notice. Because this was made after submission to PubMed, PubMed Central, and other full-text repositories, the corrected article has also been resubmitted to those repositories.

## Appendix

Multimedia Appendix 1 Originally published Figures 2 and 3.

Multimedia Appendix 2 Co-occurrence frequency of normalized post–COVID-19 condition terms in Twitter (A) which is higher than 50% and Reddit (B) which is higher than 10% data. Higher values are shown by the intensity of pink and blue shading. Normalized terms are the raw terms that were normalized (after a 2-step normalization process, as shown in Figure 1) to the 203 standardized unique concepts derived from a web-based survey of 3762 patients with post–COVID-19 condition [3]. For instance, “my tiredness” is normalized into “fatigue”.

