# Supplementary figures and images for: Using Social Media to Help Understand Patient-Reported Health Outcomes of Post–COVID-19 Condition: Natural Language Processing Approach
Source: J Med Internet Res. 2023 Sep 19;25:e45767. doi: 10.2196/45767 (PMC10510753; doi:10.2196/45767)

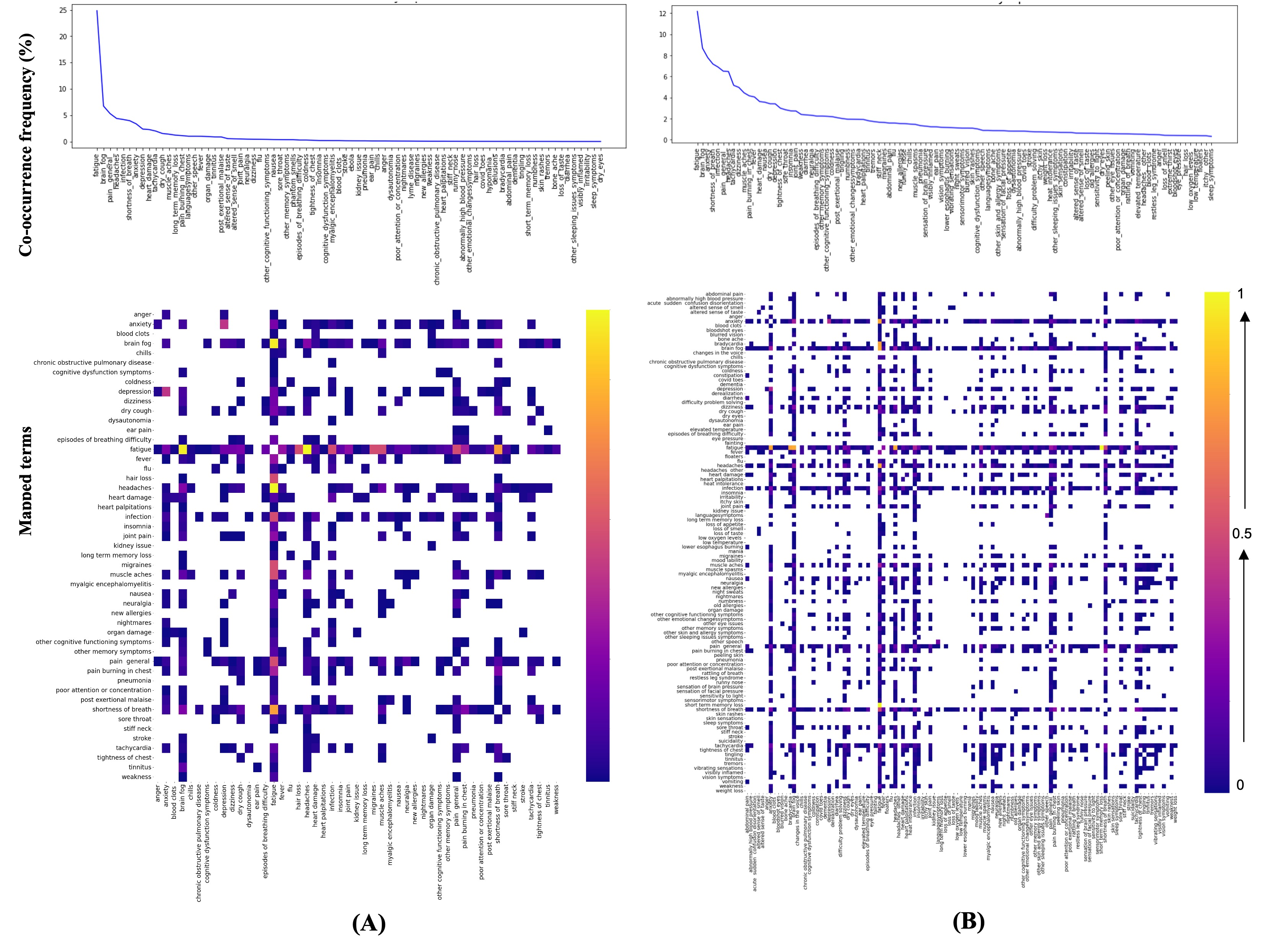

Supplement: Multimedia Appendix 2 [file jmir_v25i1e45767_app2.png]
