# Supplementary figures and images for: Figure Correction: Using Social Media to Help Understand Patient-Reported Health Outcomes of Post–COVID-19 Condition: Natural Language Processing Approach
Source: J Med Internet Res. 2023 Dec 8;25:e55010. doi: 10.2196/55010 (PMC10746960; doi:10.2196/55010)

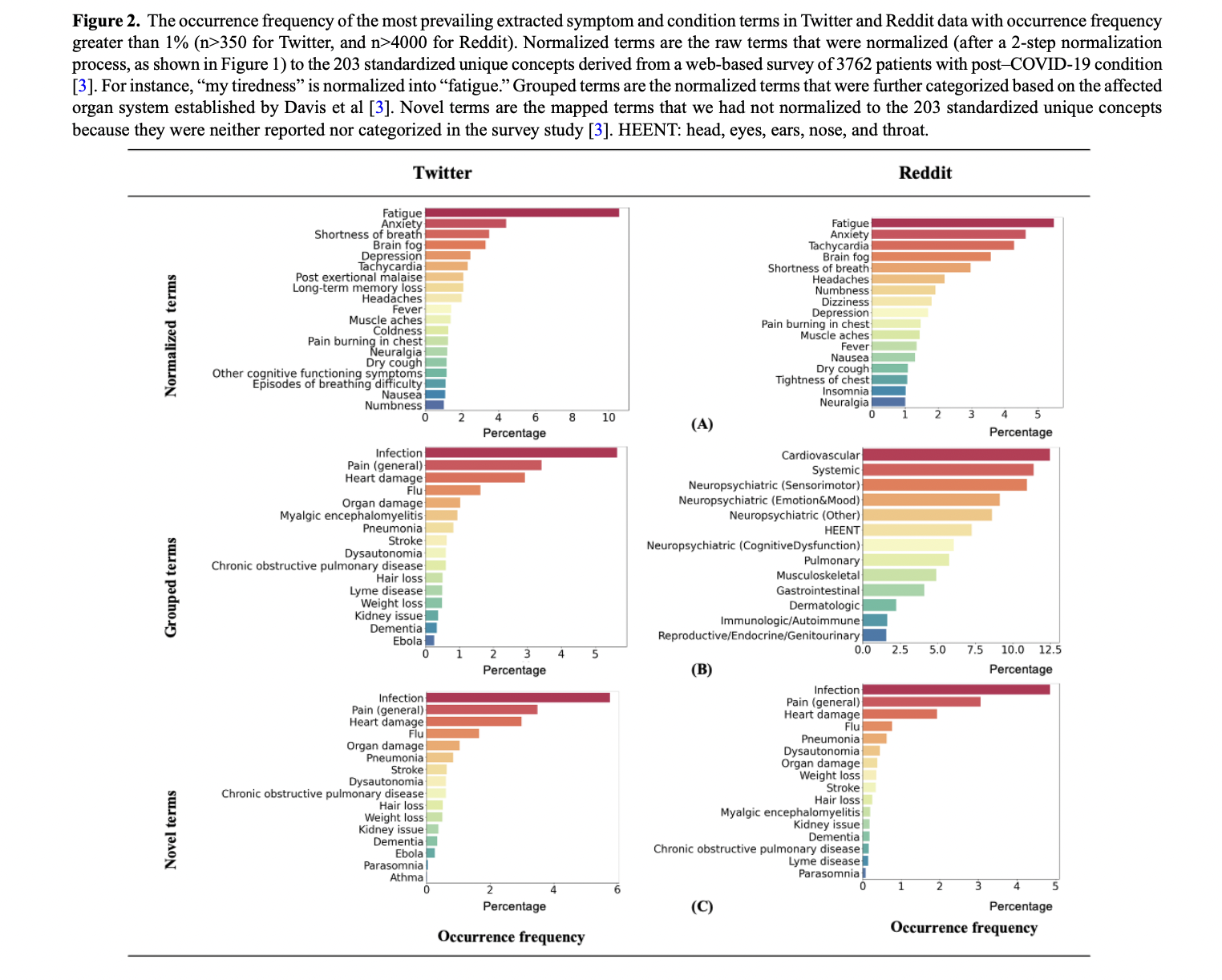

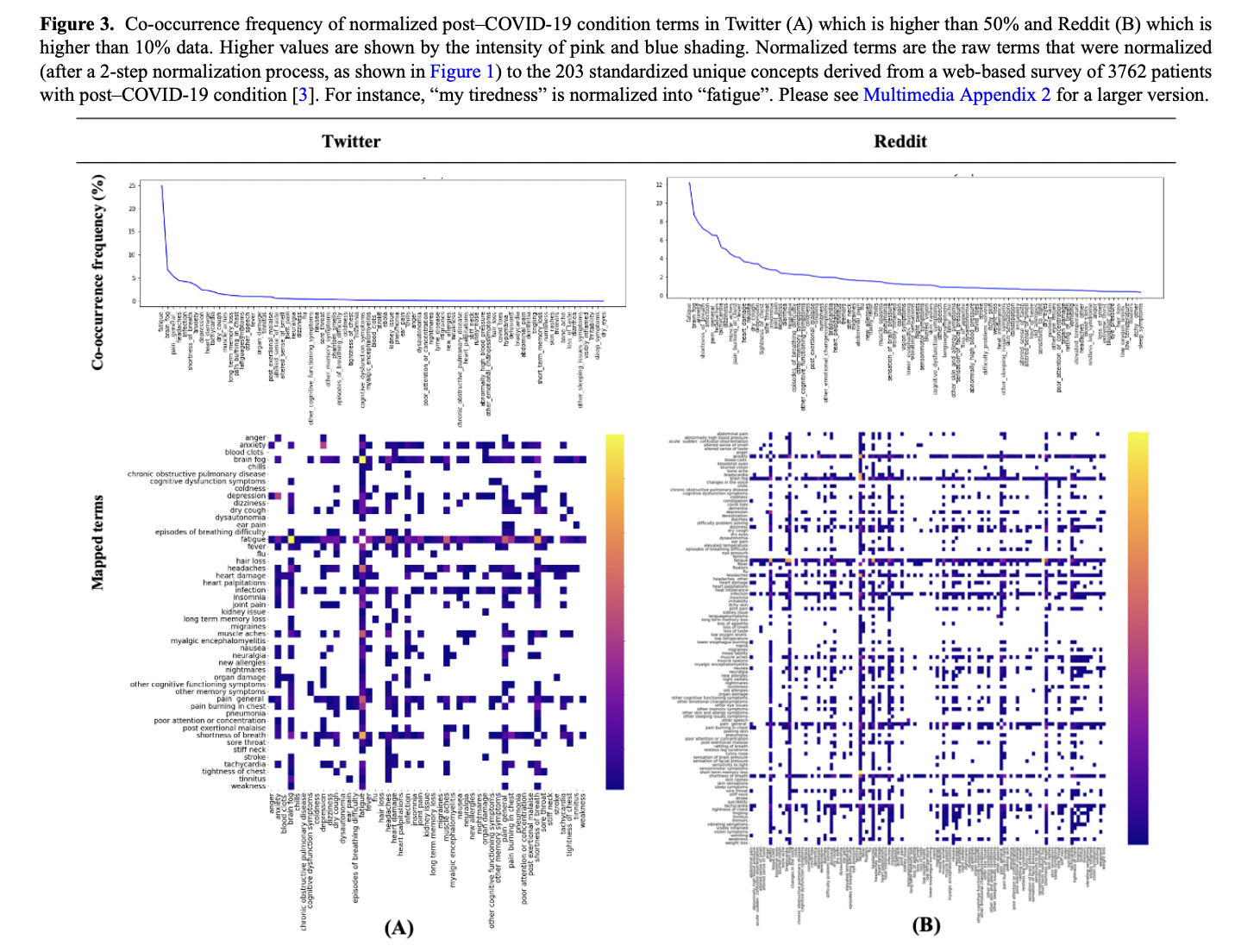

Supplement: Multimedia Appendix 1 [file jmir_v25i1e55010_app1.docx]

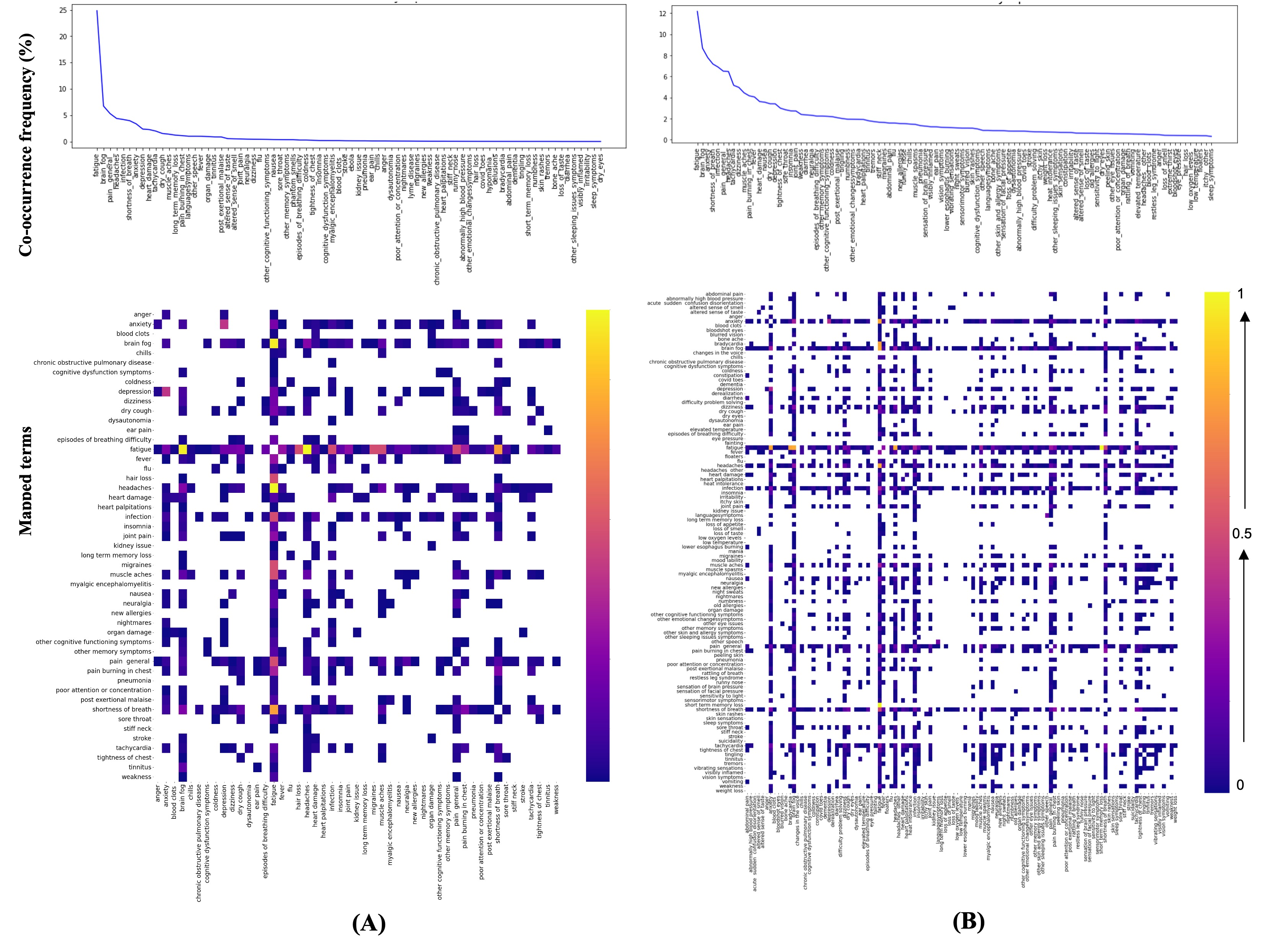

Supplement: Multimedia Appendix 2 [file jmir_v25i1e55010_app2.png]
